# Fetal loss in women exposed to cigarette smoke in-utero

**DOI:** 10.1016/j.pmedr.2025.103228

**Published:** 2025-09-01

**Authors:** Talita Honorato-Rzeszewicz, Annemieke Hoek, Maaike L. Haadsma, Henk Groen

**Affiliations:** a University of Groningen, University Medical Center Groningen, Department of Obstetrics and Gynecology, Groningen, the Netherlands; b Department of Human Genetics, Radboud University Medical Center, Nijmegen, the Netherlands; c University of Groningen, University Medical Center Groningen, Department of Epidemiology, Groningen, the Netherlands

**Keywords:** In-utero exposure, Tobacco, Cigarette smoke, Fetal loss, ALSPAC

## Abstract

**Objective:**

Animal studies have shown meiotic errors in follicles after exposure to cigarette smoke in-utero. Epidemiological studies investigating effects of in-utero smoke exposure on fetal loss in humans show inconclusive results and did not control for extraneous smoke exposure. We aimed to investigate the association between cigarette smoke exposure in-utero and risk of fetal loss, independent of active or second-hand smoking.

**Methods:**

We used data from the Avon Longitudinal Study of Parents and Children (ALSPAC) cohort (*n* = 15,445, April 1991–December 1992, Bristol, England). From obstetric history data and questionnaires completed by the participants we assessed previous fetal loss and whether the participant's mother smoked when pregnant with the participant, smoking habits and second-hand smoking. Fetal loss was defined as miscarriage up to 28 weeks of gestation or stillbirth. We performed logistic regression adjusting for confounding and stratifying for smoke exposure.

**Results:**

In 7033 women in-utero smoke exposure and fetal loss status were known; 2012 were exposed to smoke in-utero; 687 of them (34.1 %) ever had a fetal loss. Ever smokers with exposure in-utero (*n* = 1182) had higher odds of ever having a fetal loss than ever smokers unexposed in-utero (*n* = 2354, adjusted OR: 1.26; 95 % CI: 1.08, 1.47, *p*-value < 0.01 for interaction between ever smoking and in-utero exposure). Evaluation of less common combinations of in-utero and extraneous smoke exposure was hampered by small numbers.

**Conclusions:**

In-utero smoke exposure is associated with increased odds of fetal loss when occurring in combination with active smoking of the participants, compounding the odds of fetal loss.

## Highlights

• Exposure to cigarette smoke in utero has been shown to result in damage to oocytes.
• Such damage may result in adverse pregnancy outcomes such as fetal loss.
• We used data from the Avon Longitudinal Study to examine this hypothesis.
• Cigarette smoke exposure in utero was associated with a higher risk of fetal loss.
• This risk was compounded by smoking in later life.

## 1 Introduction

In most European countries the incidence of smoking during pregnancy decreased in the last decades (Euro-Peristat Project, 2018). However, Europe still has the highest prevalence of smoking during pregnancy (8.1 %; 95 % CI: 4.0–12.2) compared to the global prevalence (1.7 %; 95 % CI: 0.0–4.5) (Lange et al., 2018).

Smoking affects folliculogenesis, steroidogenesis, embryo transport through the fallopian tubes, embryo implantation, and myometrial activity (Richardson et al., 2014; Dechanet et al., 2011; Yao et al., 2021). It has been hypothesized that exposure to cigarette smoking in-utero has a negative effect on the reproductive capacity of the unborn female offspring (Yasui et al., 2012). In females, reproductive capacity is mainly established during the embryonic and fetal period (Grive and Freiman, 2015; Kermack et al., 2015), during which the female germ cells are vulnerable to harmful agents such as cigarette smoke (Dechanet et al., 2011; Fowler et al., 2014; Camlin et al., 2016; Paccola et al., 2021). Previous studies showed that women who were exposed in-utero had longer time to pregnancy (Jensen et al., 1998; Jensen et al., 2006), altered hormone levels around puberty, and lower age at menarche (Ernst et al., 2012; Gollenberg et al., 2015) and menopause (Langton et al., 2022) compared to unexposed women.

Evidence from mice, rats and human studies suggests that in-utero smoke exposure might reduce the number and the quality of female germ cells (Camlin et al., 2016; Lutterodt et al., 2009). Mice exposed in-utero had meiotic errors in oocytes due to alterations in spindle formation (Camlin et al., 2017). In humans, meiotic errors in oocytes could lead to increased odds of fetal loss, especially in early stages of embryo development (Eichenlaub-Ritter, 2012). Experimental mice studies have the disadvantage that mice complete their oocyte pool perinatally, which is different from humans. Previous epidemiological human studies did not provide a clear answer whether in-utero smoke exposure is associated with higher odds of fetal loss. Importantly, these studies did not control for other sources of smoke exposure, which could have blurred the isolated effect of in-utero smoke exposure. It is not known whether women who are non-smokers but were exposed to cigarette smoke in-utero have higher odds of fetal loss compared with women who are neither smokers nor were exposed in-utero.

Therefore, the aim of our study was to explore whether in-utero exposure to cigarette smoke is associated with higher odds of fetal loss later in life, independently of other sources of smoke exposure. We used data from the UK Avon Longitudinal Study of Parents and Children (ALSPAC) cohort, a unique and large database containing transgenerational information of cigarette smoke exposure in-utero and in later life.

## 2 Materials and methods

### 2.1 Participants

The ALSPAC study was established to investigate environmental and genetic factors that could potentially influence the health of parents and children (Boyd et al., 2013; Golding, 1990). The full description of the ALSPAC cohort has been previously reported (Fraser et al., 2013). Briefly, all pregnant women (the participants, subjects of this study) residing in the Avon County around Bristol, England, with expected date of delivery for the index child between 1st April 1991 and 31st December 1992 were invited to enrol in the study. Only one index pregnancy was included for each participating woman. Initially, 15445 women (i.e. approximately 90 % of all pregnancies in the study area for the enrolment period) were enrolled in the study. Participants were recruited by local press (radio, television, posters), local community midwives and during routine ultrasound examinations. Questionnaires were pilot tested previously and validity of the questionnaires was assessed by comparing answers regarding the participants' obstetric history, prescribed medication and morbidity with their medical records. The agreement was found to be 95 % (Golding et al., 2001).

#### 2.1.1 Fetal loss

Characteristics and obstetric history of the participants were obtained from medical records and questionnaires filled in during the gestational period of the index child. The obstetric history included self-reported information regarding previous miscarriages, abortions or terminations, stillbirths, perinatal deaths and birth weight and term of delivery. When pregnant with the index child, participants were asked “were you pregnant before? if yes, how many times?” and “did you ever have a miscarriage? if yes how many times?” Information obtained from medical records indicated that participants who had a miscarriage had a pregnancy loss up to 28 weeks of gestation. Participants were also asked “have you ever had a stillbirth?”. We defined fetal loss as at least one event of miscarriage or stillbirth, irrespective of gestational age.

The ALSPAC study aimed to follow parents and children resulting from the index pregnancy. Therefore, most of the participants whose index pregnancy resulted in a miscarriage were excluded from further assessments. Subsequently, we did not analyse the outcome of the index pregnancy, which were exclusively live births, but we assessed the preceding pregnancies to avoid selection bias. The obstetric history of the participants analyzed for this study comprises the period between the first and last pregnancy preceding the index pregnancy.

#### 2.1.2 In-utero *smoke exposure*

Questions regarding in-utero cigarette smoke exposure were answered by the participants at two different time points: when participants were pregnant with the index child and eight years later. Participants were asked: “did your mother smoke when she was expecting you” and participants could answer: “yes”, “no” or “don't know”. Answers from both time points were compared and inconsistent answers (changes from “yes” to “no” or vice-versa) were excluded. Participants who answered “don't know” in both time points were also excluded. Participants who at first answered “don't know” but answered “yes” or “no” eight years later were reclassified, assuming that they retrieved the information, e.g. from their mothers after completing the first questionnaire. The consistency between responses from two time points was high (Cohen's Kappa 0.88 with *n* = 2550, *p* = 0.01). Sensitivity analyses without reclassification of in-utero exposure of the participants were performed to evaluate any differences in results due to reclassification.

#### 2.1.3 Other sources of smoke exposure

Besides in-utero smoke exposure, we also considered active smoking and second-hand smoking. Active smoking refers to the smoking habits of participants themselves, dichotomizing participants into never smokers or ever (i.e. current or previous) smokers. When pregnant with the index child, participants were asked “have you ever been a smoker?”, “what age did you start smoking regularly?”, “what was the maximum number of times a day you smoked?”, “have you stopped smoking. If yes, how long?” We did not calculate cumulative exposure, i.e. average daily packs of cigarettes times years of exposure (pack-years), since we could not determine the number of pack-years before fetal loss. Second-hand smoking was defined as exposure of the participants to smoking by their father, partner or other household members. When pregnant with the index child, participants were asked “did your father ever smoke?”, “does your partner smoke?” and “ are there any other members of your household who smoke?”

### 2.2 Statistical analysis

Our hypothesis that in-utero smoke exposure is associated with higher odds of a fetal loss, was tested by dividing the participants into groups according to exposure to cigarette smoke in-utero. Differences in characteristics between exposed and non-exposed participants were assessed using independent samples Student's *t*-test for continuous variables or Chi-square test for categorical variables. If the Levene's test for equality of variances had a *p*-value <0.05, the Mann-Whitney *U* test was used for continuous variables. All characteristics available were tested to check if they were associated with the odds of having a fetal loss with logistic regression analysis. In the analysis of fetal loss depending on in-utero smoke exposure we adjusted for confounding by characteristics associated with fetal loss that also differed between participants who were exposed or not exposed in-utero. To assess the possibility of bias due to selection of participants with available data for all variables in the adjusted analyses, the unadjusted odds ratio was also calculated in the same population as the adjusted analysis. Finally, interaction between in-utero exposure and other sources of smoke exposure was explored by adding interaction terms to the regression analysis.

A *p*-value<0.05 was considered statistically significant. Analyses were performed using SPSS Statistics, version 26 (IBM Corporation, Armonk, NY, USA).

Please note that the study website contains details of all the data that are available through a fully searchable data dictionary available at http://www.bris.ac.uk/alspac/researchers/data-access/data-dictionary. Ethical approval for the study was obtained from the ALSPAC Ethics and Law Committee and the Local Research Ethics Committees available at http://www.bristol.ac.uk/alspac/researchers/research-ethics. Informed consent was obtained from all individual participants included in the study.

## 3 Results

### 3.1 Description of the participants

**Fig. 1:**
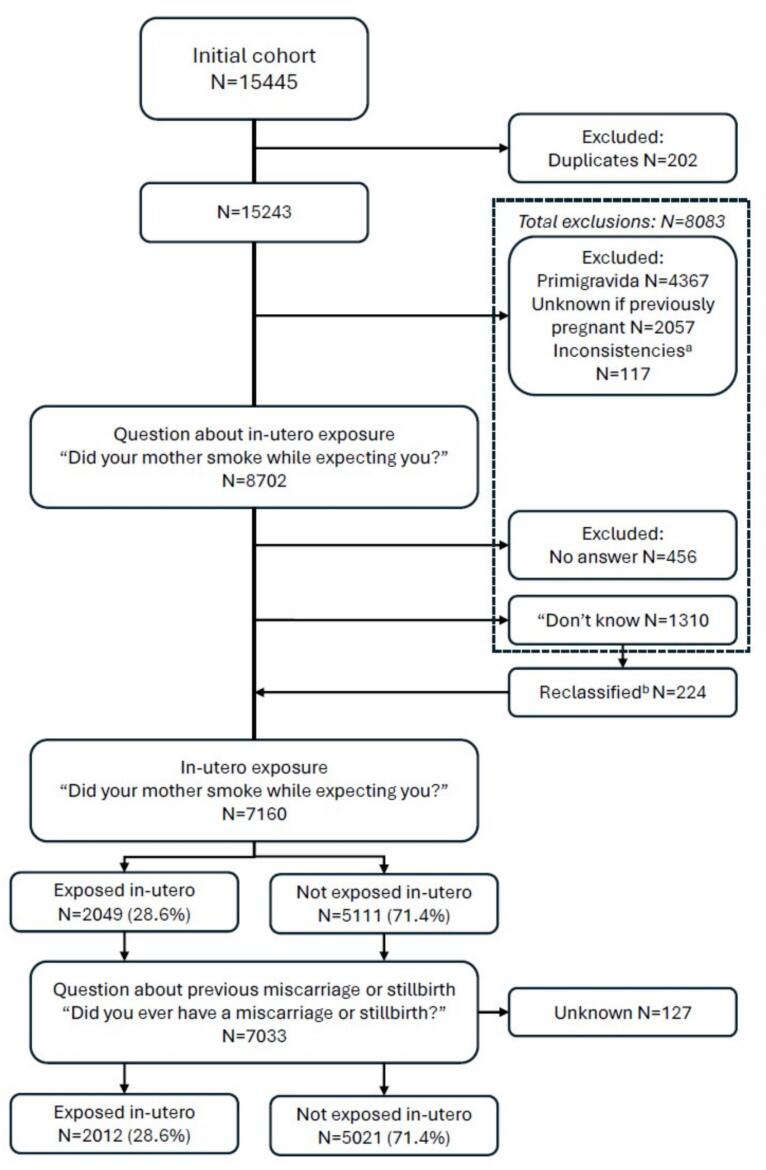
Flowchart of pregnant women selected from the Avon Longitudinal Study Parents and Children cohort (Bristol, England, 1991–1992). Legend: a: Participants with incompatible number of miscarriages per number of pregnancies b. Participants who answered ‘Don't know’ to the question about in-utero exposure in the first questionnaire, but indicated they were exposed (*n* = 87) or not exposed (*n* = 137) in a later questionnaire were reclassified and included in the study.

The initial cohort included 15,243 unique participants. After exclusions, mainly due to primigravidity and unknown previous pregnancy (total *n* = 8083, see Fig. 1), 7160 participants were included in the analyses, of whom 28.6 % (2049/7160) were exposed to cigarette smoke in-utero. Information regarding fetal loss was not available in 127 (1.77 %) of these women, 37/2049 (1.81 %) in women with in-utero exposure and 90/5111 (1.76 %) in women not exposed in utero. Among the 2279/7033 (32.4 %) participants who had a fetal loss, 2230 (97.8 %) had at least one miscarriage and 89 (3.91 %) had at least one stillbirth. Of the latter, 40 women also had at least one miscarriage.

Table 1 shows characteristics of the participants and the differences between groups of in-utero exposed and non-exposed participants. In-utero exposed participants were, on average, younger at first pregnancy (22.1 ± 4.6 years of age versus 23.9 ± 4.7) and at last pregnancy (median 27.0 versus 28.0), had more pregnancies (median 2 versus 1), were slightly more often underweight (5.77 % versus 4.78 %) or obese (7.19 % versus 5.20 %), had a lower educational level (Certificate of Secondary Education level 25.4 % versus 12.6 %) and more often had diabetes (1.80 % versus 0.89 %). Overall, the proportion of ever smokers (current or previous smokers) was high in both groups (60.2 % and 47.7 %) but compared to unexposed, in-utero exposed participants started smoking earlier (15.8 + 2.8 years of age versus 16.6 ± 2.8), were more often current smokers (47.1 % versus 29.0 %), and more often had fathers (89.4 % versus 70.3 %), partners (47.1 % versus 35.3 %) or other household members who smoked (6.71 % versus 3.62 %).

**Table 1:** Comparison of characteristics of women with known in-utero exposure to cigarette smoke, exposed versus unexposed, in the ALSPAC* cohort (Bristol, England, 1991–1992).

Characteristic	N	Exposed in-utero *n* = 2049/7160 (28.6 %)	Not exposed in-utero *n* = 5111/7160 (71.4 %)	p-value
Number of previous pregnancies (median, range)	7104	2.0 (1−11)	1.0 (1−13)	<0.01^e^
1		897/2028 (44.2)	2589/5076 (51.0)	<0.01^f^
2		550/2028 (27.1)	1418/5076 (27.9)	
3		310/2028 (15.3)	658/5076 (13.0)	
4 or more		271/2028 (13.4)	411/5076 (8.04)	
Age at first pregnancy (mean, SD, range)	7156	22.1 ± 4.64 (12–40)	23.9 ± 4.65 (10–40)	<0.01^g^
≤20 years		886/2047 (43.3)	1320/5109 (25.8)	<0.01^f^
21–35 years		1146/2047 (56.0)	3750/5109 (73.3)	
≥36 years		15/2047 (0.73)	39/5109 (0.76)	
Age at last pregnancy (median, range)	7160	27.0 (15–44)	28.0 (15–44)	<0.01^e^
≤20 years		187/2049 (9.13)	169/5111 (3.31)	<0.01^f^
21–35 years		1731/2049 (84.5)	4601/5111 (90.0)	
≥36 years		131/2049 (6.39)	341/5111 (6.67)	
Interval between 1st and last pregnancy (years, median, range)	7155	4.0 (0–26)	3.0 (0–26)	<0.01^e^
Interval between 1st and last pregnancy by maternal age category (years, median, range)				
≤20 years	7155	6.0 (0–24)	7.0 (0–26)	<0.01^e^
21–35 years		3.0 (0−21)	3.0 (0−22)	0.02^e^
≥36 years		2.0 (0–3)	1.0 (0–4)	0.59^e^
Active smoking of participants	6981			<0.01^f,h^
Never smoker		794/1994 (39.8)	2608/4987 (52.3)	
Ever smoker		1200/1994 (60.2)	2379/4987 (47.7)	
Current smoker		939/1994 (47.1)	1444/4987 (29.0)	
Previous smoker		261/1994 (13.1)	935/4987 (18.7)	
Age started smoking (mean, SD)	3695	15.8 ± 2.8	16.6 ± 2.8	<0.01^g^
Years since stop smoking (mean, SD)	1196	4.9 ± 3.2	5.3 ± 3.3	0.039^g^
Passive smoking of participants				
Participant's mother is a smoker	7160	2049/2049 (100)	1581/5111 (30.9)	NA
Participant's father is a smoker^a^	6966	1767/1977 (89.4)	3505/4989 (70.3)	<0.01^f^
Participant's partner is a smoker^a^	6927	930/1973 (47.1)	1745/4944 (35.3)	<0.01^f^
Other household members are smokers^a^	6995	134/1998 (6.71)	181/4997 (3.62)	<0.01^f^
Level of education	6158			<0.01^f^
CSE^b^		423/1663 (25.4)	566/4495 (12.6)	
Vocational		198/1663 (11.9)	458/4495 (10.2)	
O level^c^		594/1663 (35.8)	1665/4495 (37.0)	
A level^d^		326/1663 (19.6)	1120/4495 (24.9)	
Degree		122/1663 (7.34)	686/4495 (15.3)	
BMI (kg/m^2^, median, range)^a^	6142	22.5 (14.9–47.7)	22.2 (14.2–47.3)	<0.01^e^
≤18.5 Underweight	6142	97/1682 (5.77)	213/4460 (4.78)	<0.01^f^
18.6–24.9 Normal		1174/1682 (69.8)	3307/4460 (74.1)	
25–29.9 Overweight		290/1682 (17.2)	708/4460 (15.9)	
≥30 Obese		121/1682 (7.19)	232/4460 (5.20)	
History of diabetes	6569	33/1829 (1.80)	42/4740 (0.89)	<0.01^f^
Insulin dependent		9/24 (37.5)	15/37 (40.5)	
Diet only		13/24 (54.2)	22/37 (59.5)	
Alcohol consumption this pregnancy^a^	7123	1852/2037 (90.9)	4616/5086 (90.8)	0.01^f^
Birth weight of the participant (median, range)	4129	3167.6 (909.1–5113.6)	3352.3 (909.1–6108.0)	<0.01^e^

Footnote: *: Avon Longitudinal Study of Parents and Children (ALSPAC) study from Bristol, England. Questionnaire assessment year: 1992. Numbers are n/N (percentage) by exposure group unless indicated otherwise. **a**. Assessed at index pregnancy **b**. Certificate of Secondary Education **c**. General certificate of education “ordinary” level **d**. General certificate of education “advanced” level. **e**. Mann-Whitney U Test **f**. Pearson Chi-Squared Test **g**. Independent samples T-test **h**. Current, previous and never smokers included. NA: not applicable, variable directly related to in-utero exposure.

#### 3.1.1 Potential confounders

Table 2 shows unadjusted ORs of potential confounders for the effect of in-utero exposure on fetal loss. These factors were: longer interval between first and last pregnancies (OR 1.04 95 % CI: 1.02, 1.04) and older age at first (OR 2.61, 95 % CI: 1.51, 4.50) and last pregnancy (OR 1.84, 95 % CI: 1.52, 2.22), current smoking (OR 1.11, 95 % CI: 0.99, 1.24), years since discontinuation of smoking (OR 1.04, 95 % CI: 1.01, 1.08), second-hand smoking exposure from smoking of the father (OR 1.18, 95 % CI: 1.05, 1.33) or of other household members (OR 1.45, 95 % CI: 1.15, 1.83).

**Table 2:** Univariable analysis of potential confounders of the effect of in-utero exposure to cigarette smoke on the odds of fetal loss in women from the ALSPAC* cohort (Bristol, England, 1991–1992).

Variable	N	OR (95 % CI)
Age at first pregnancy	7029	1.00 (0.99, 1.01)
≤20 yrs		1.09 (0.98, 1.22)
21–35 yrs		Reference
≥36 yrs		2.61 (1.51, 4.50)
Age at last pregnancy	7033	1.03 (1.02, 1.04)
≤20 yrs		0.99 (0.78–1.26)
21–35 yrs		Reference
≥36 yrs		1.84 (1.52, 2.22)
Interval between first and last pregnancy	7028	1.04 (1.02, 1.05)
Active smoking of participants	6873	
Never smoker		Reference
Current		1.11 (0.99, 1.24)
Previous		1.04 (0.90, 1.20)
Age started smoking	3635	1.02 (0.99, 1.04)
Years since stop smoking	1569	1.02 (0.99, 1.05)
Passive smoking of participants		
Participant's father is a smoker	6845	1.18 (1.05, 1.33)
Participant's partner is a smoker	6857	1.05 (0.95, 1.17)
Other household members are smokers	6936	1.45 (1.15, 1.83)
Level of education	6049	
CSE^a^		Reference
Vocational		0.82 (0.66, 1.01)
O level^b^		0.86 (0.73, 1.01)
A level^c^		0.94 (0.79, 1.11)
Degree		0.94 (0.77, 1.15)
BMI (kg/m^2^)	6038	1.02 (1.00, 1.03
≤18.5 Underweight		0.96 (0.74, 1.23)
18.6–24.9 Normal		Reference
25–29.9 Overweight		1.07 (0.92, 1.24)
≥30 Obese		1.10 (0.87, 1.39)
Diabetes	6456	1.44 (0.89, 2.31)
Alcohol consumption this pregnancy^d^	6999	0.93 (0.78, 1.10)
Birth weight of the participant (kg)	4062	0.94 (0.84, 1.04)

Footnote: *: Avon Longitudinal Study of Parents and Children (ALSPAC) study from Bristol, England. Questionnaire assessment year: 1992. **a**. Certificate of Secondary Education **b**. General certificate of education Ordinary level **c**. General certificate of education advanced level. **d**. Assessed at index pregnancy. OR: Odds ratio. CI: confidence interval.

In the multivariable analyses we therefore adjusted for interval between and age at first and last pregnancy, and passive exposure to smoking by father and other household smokers. Although current smoking and, for previous smokers, higher number of years since smoking discontinuation were also associated with fetal loss, we did not adjust our analyses for these variables since these were highly correlated and could introduce overadjustment bias, conditioned to the shared effect of these variables (Schisterman et al., 2009).

#### 3.1.2 Odds of fetal loss

Table 3 shows ORs of ever having a fetal loss associated with in-utero exposure. The unadjusted overall effect of in-utero exposure, without considering other sources of cigarette smoke exposure, was small but significant (unadjusted OR 1.12, 95 % CI: 1.00, 1.25). The adjusted odds ratio was similar but not statistically significant (aOR 1.10, 95 % CI: 0.99, 1.24). Sensitivity analysis without the reclassified participants yielded similar results (unadjusted OR 1.12, 95 % CI: 1.00, 1.25; aOR 1.10, 95 % CI: 0.98, 1.24).

**Table 3:** Association between in-utero exposure to cigarette smoke and the odds of ever having a fetal loss in the ALSPAC* cohort (Bristol, England, 1991–1992).

Fetal loss by group		n/%^a^	OR (95 % CI)	
In-utero exposed (*n* = 2012)	Not in-utero exposed (*n* = 5021)			
Fetal loss in all women, independently of smoking status or second-hand smoke exposure:	Fetal loss in all women, independently of smoking status or second-hand smoke exposure:	7033/98.2	Unadjusted	1.12 (1.00, 1.25)
687/2012, 34.1 %^b^ (95 % CI: 32.1; 36.2)	1592/5021, 31.7 % (95 % CI: 30.4; 33.0)	6724/93.9	Adjusted	1.11 (0.99, 1.25)^c^
		6724/93.9	Unadjusted*	1.13 (1.01, 1.26)
Stratified by smoking status				
In-utero exposed (*n* = 1963)	Not in-utero exposed (*n* = 4910)	p-value interaction:< 0.01 (unadjusted)/<0.05 (adjusted)		
Fetal loss in never smokers:	Fetal loss in never smokers:	3337/46.6	Unadjusted	0.91 (0.76, 1.08)
233/781, 29.8 % (95 % CI: 26.6; 33.0)	814/2556, 31.8 % (95 % CI: 30.0; 33.6)	3246/45.3	Adjusted	0.92 (0.76, 1.10)^d^
		3246/45.3	Unadjusted*	0.93 (0.78, 1.11)
Fetal loss in ever smokers:	Fetal loss in ever smokers:	3536/49.4	Unadjusted	1.26 (1.09, 1.46)
433/1182, 36.6 % (95 % CI: 33.9; 39.4)	739/2354, 31.4 % (95 % CI: 29.5; 33.3)	3478/48.6	Adjusted	1.28 (1.10, 1.49)^d^
		3478/48.6	Unadjusted*	1.27 (1.09, 1.47)
Stratified by smoking status and second-hand smoke exposure				
In-utero exposed (*n* = 1938)	Not in-utero exposed (*n* = 4791)	p-value interaction: 0.08 (unadjusted)/0.05 (adjusted)		
Fetal loss in never smokers, not exposed to second-hand smoke:	Fetal loss in never smokers, not exposed to second-hand smoke:	749/10.5	Unadjusted	0.40 (0.20, 0.81)
10/67, 14.9 % (95 % CI: 5.6; 20.8)	206/682, 30.2 % (95 % CI: 26.8; 33.6)	749/10.5	Adjusted	0.41 (0.20, 0.80)^e^
			Unadjusted*	NA
Fetal loss in never smokers, exposed to second-hand smoke:	Fetal loss in never smokers, exposed to second-hand smoke:	2498/34.9	Unadjusted	0.97 (0.81, 1.17)
219/700, 31.3 % (95 % CI: 27.8; 34.7)	573/1798, 31.9 % (95 % CI: 29.7; 34.0)	2497/34.9	Adjusted	0.99 (0.82, 1.20)^e^
		2497/34.9	Unadjusted*	0.97 (0.81, 1.18)
		p-value interaction: 0.61 (unadjusted)/0.68 (adjusted)		
Fetal loss in ever smokers, not exposed to second-hand smoke:	Fetal loss in ever smokers, not exposed to second-hand smoke:	331/4.62	Unadjusted	1.49 (0.76, 2.91)
16/43, 37.2 % (95 % CI: 22.8; 51.7)	82/288, 28.5 % (95 % CI: 23.3; 33.7)	330/4.61	Adjusted	1.61 (0.80, 3.23)^e^
		330/4.61	Unadjusted*	1.48 (0.76, 2.89)
Fetal loss in ever smokers, exposed to second-hand smoke:	Fetal loss in ever smokers, exposed to second-hand smoke:	3151/44.0	Unadjusted	1.24 (1.07, 1.45)
415/1128, 36.8 % (95 % CI: 34.0; 39.6)	645/2023, 31.9 % (95 % CI: 29.8; 33.9)	3148/44.0	Adjusted	1.26 (1.08, 1.47)^e^
		3148/44.0	Unadjusted*	1.24 (1.06, 1.45)

Footnote: *:Avon Longitudinal Study of Parents and Children (ALSPAC) study from Bristol, England. Questionnaire assessment year: 1992. Unadjusted*: unadjusted analysis with same cases as adjusted; **a.** number and percentage of participants included in the analysis, relative to overall *N* = 7160 **b.** percentage of fetal loss **c**. adjusted for interval and age at last and first pregnancy, ever smoking, and second-hand smoking **d**. adjusted for interval between and age at last and first pregnancy, and second-hand smoking **e**. adjusted for interval between and age at last and first pregnancy. NA: not applicable. OR: Odds ratio. CI: confidence interval.

#### 3.1.3 Stratified analysis for smoking habits for never smokers and ever smokers

We performed stratified analyses according to smoking habits to examine the effects of in-utero exposure in never smokers and ever smokers. For never smokers, in-utero exposed participants did not have a significantly different odds of ever having a fetal loss compared with unexposed participants (aOR 0.91, 95 % CI: 0.76, 1.10). On the other hand, for ever smokers, in-utero exposed participants had higher odds of fetal loss compared to non in-utero exposed participants (aOR 1.26, 95 % CI: 1.08, 1.47). The difference in effect in ever smokers and never smokers was statistically significant (*p*-value <0.01 for interaction between in-utero smoke exposure and ever smoking).

#### 3.1.4 Stratified analyses by second-hand smoke exposure

Due to the small numbers of participants who were not exposed to second-hand smoke, strata for never smokers and ever smokers without second-hand smoke exposure were only 10.5 % and 4.62 % of our population, respectively. More specifically, the groups of in-utero exposed women who were never smokers and were not exposed to second-hand smoke (*n* = 67, 0.90 % of the population) and in-utero exposed women who were ever smokers but not exposed to second-hand smoke (*n* = 43, 0.58 %) were extremely small. As a result, the 95 % confidence intervals of the outcome rates in these groups were very wide. The results suggest a reduction of fetal loss due to in-utero exposure in women who were not otherwise exposed to any smoke, but the effect was not significantly different from women who were never smokers but exposed to second-hand smoke. In ever smokers, the effect of in-utero exposure combined with second-hand smoke exposure was more pronounced than in women without second-hand smoke exposure, but the difference in effect was not significant.

## 4 Discussion

The aim of our study was to explore whether in-utero exposure to cigarette smoke is associated with higher odds of fetal loss later in life, independently of other sources of smoke exposure. Our unadjusted analysis showed that in-utero smoke exposure slightly increased the odds of fetal loss. After adjusting for other sources of cigarette smoke exposure the odds ratio was similar in size but not significant. This overall result is similar to previous epidemiological studies in which other sources of smoke exposure were not analyzed separately. One previous study, including 76.357 pregnancies from 1999 to 2008 from the Norwegian Medical Birth Registry and reporting 59 late miscarriages (between 17 and 20 weeks gestational age) (Cupul-Uicab et al., 2011) found a non-significantly increased risk of late miscarriage in women exposed to cigarette smoke in-utero (HR 1.23, 95 % CI: 0.72, 2.12). The rate of in-utero cigarette smoke exposure in the Norwegian data was 28 %, which is comparable to 28.6 % in-utero exposure in our cohort. Another study, with a 31 % in-utero smoke exposure rate in a large dataset of 12,321 participants, showed that women who were in-utero exposed to smoke were more likely to experience a single or repeated miscarriage (OR 1.16, 95 % CI 1.01–1.32) but were also more likely to experience a pregnancy in general (aOR 1.25, 95 % CI 1.13, 1.38) compared to non-exposed women (Tweed et al., 2017).

Our results also showed that the effect of in-utero smoke exposure was different for never smokers compared to ever smokers. Ever smokers who were exposed in-utero had higher odds of fetal loss compared to ever smokers who were not exposed in-utero. This difference in effect was statistically significant, demonstrating interaction between in-utero smoke exposure and lifetime exposure to cigarette smoking. Interaction suggests a biological mechanism by which in-utero smoke exposure influences the effect of ever smoking (or vice versa), compounding the odds of fetal loss.

Evaluation of less common combinations of in-utero exposure and smoking habits was hampered by small numbers. Due to the high prevalence of smoking in partners and family members, strata for never smokers and ever smokers without second-hand smoke exposure were very small. Therefore, these analyses were not considered to be sufficiently reliable to allow any conclusions. Despite our efforts to adjust for confounding, apparent effects may be influenced by residual confounding related to the intricacies of associations between socioeconomic determinants and smoking in this population.

This study contributes to the scarce literature regarding in-utero cigarette smoke exposure and odds of fetal loss. The ALSPAC cohort is the only source we know of that has the information required to test our hypothesis. Although the data originate from some time ago it suits the purpose of our study, having sufficiently large exposure to find an effect. However, with the current prevalence of smoking during pregnancy effects may be weaker. Strengths of this study include the large sample size, assessment of various important potential confounders, low chance of selection bias due to small difference of in-utero smoke exposure between participants included and excluded in the analyses. The questionnaires were previously internally validated with pilot testing to guarantee clear questions and to avoid confusion in responses. Responses to items from the questionnaires that were also recorded in medical records were compared, showing a 95 % agreement rate. To avoid misclassification and reduce recall bias for in-utero exposure, agreement between answers to questionnaires at enrolment and eight years later was assessed and showed high level of agreement, meaning that the majority of the participants were consistent in their answers. Indirect evidence for in-utero smoke exposure was found in the comparison between birth weights of in-utero exposed and not-exposed participants (3109 g versus 3326 g respectively, *p*-value <0.01), showing lower average birth weight in in-utero exposed participants. Additionally, life course cigarette smoke exposure from childhood into adolescence, which was not considered in previous studies, was addressed indirectly in this study based on parental smoking habits.

An important limitation of our study may be the fact that only women who achieved an ongoing pregnancy were included in the ALSPAC cohort. The absence of women whose oocyte quantity or quality was affected to the degree that they could not sustain a pregnancy to term and were prone to fetal loss could potentially underestimate the effect of in-utero exposure. Further limitations of this study include unknown gestational age, lifestyle characteristics, smoking status and cumulative smoke exposure, expressed as packyears, at the time of the fetal loss. Therefore gestational age at which the highest proportion of fetal loss might have occurred as well as the dose-dependence of the effect of smoke exposure remains unknown. Similarly, we could not explore dose-dependence of in-utero smoke exposure since the number of cigarettes smoked per day by the mother of the participants was unknown. Finally, despite the large number of participants in the initial cohort, the number of women with the lowest smoke exposure were quite small, which precluded analysis of the effects in these groups. Even in large cohorts, disentangling the effects of various sources of exposures can be difficult. In-utero smoke exposure is associated with nicotine dependence in the offspring (Cornelius et al., 2000; Oncken et al., 2004). Indeed, women who were in-utero exposed were more likely to be smokers in our population. Additionally, women of lower educational level had higher chances of being exposed to cigarette smoke in-utero and social determinants are known for being associated with poor health outcomes. In our study we could not explore social determinants; the majority of the participants were white (97 %) and had at least ordinary level of education (73 %). Finally, due to the retrospective study design, the lifestyle characteristics of participants collected during the pregnancy of the index child were taken to adjust estimates for fetal loss occurring prior to the index pregnancy. Specifically, we tried to assess the effects of age of the participants and smoking status in two ways. Firstly, since the risk of fetal loss increases with advancing age, we adjusted for age at first and last pregnancy as well as for interval between first and last pregnancy. The adjustment did not materially change the results which remained statistically significant. Secondly, we took smoking status of participants at the time of enrolment as a proxy for smoking status when fetal loss occurred because these data were not available for previous pregnancies. We were able to establish that 216 participants started smoking after the age of their first pregnancy. These participants were all multiparas and none of them started smoking after the age of the last pregnancy. Analyses excluding these participants did not affect the results significantly. The Annual Population Survey in Great Britain has shown a downward trend in smoking since 1974. The most recent survey in 2021 reveals 13.3 % smokers, a decrease by 6.9 percentage points compared to 2011 (Office for National Statistics: Adult smoking habits in the UK, 2021). In our data, the largest average interval between first and last pregnancy was 7.6 years, therefore we could expect a maximum of 5 % change in smoking behavior of participants, which is not likely to have a major impact on our results.

There have been several animal studies showing possible detrimental biological effects of in-utero exposure to cigarette smoke on quality and quantity of oocytes (Camlin et al., 2016; Langton et al., 2022). Early exposure may program autophagy of oogonia, leading to premature or excessive cell death in oocytes triggered by persistent injury such as active of passive smoking during life (Lockshin and Zakeri, 2004; Debnath et al., 2005). This would not only diminish the follicle pool, the quality of the remaining follicles could also compromised (Johansson et al., 2017). These biological mechanisms can neither be confirmed or refuted in epidemiologic studies such as ours, but our results are in line with the hypothesis that women who were exposed to cigarette smoke in-utero and are smokers might have a diminished follicle pool and lower quality follicles, as expressed in an increased risk of fetal loss. In support of this hypothesis, previous work from our group and others has shown that smokers exposed to cigarette smoke in-utero had higher hazards of earlier menopause compared to smokers who were not exposed in-utero (HR after adjustment: 1.93, 95 % CI: 1.25, 2.99) (Langton et al., 2022; Honorato et al., 2018).

In conclusion, our study showed that in-utero smoke exposure increased the odds of fetal loss in women who were exposed to other sources of smoke during life. Compared to participants who were not exposed in utero but were smokers, participants who were exposed in-utero and were smokers had higher odds of fetal loss. Future studies are required to validate our results in other populations and to disentangle the interrelationships between in-utero smoke exposure, smoking, and second-hand smoke exposure and other socioeconomic determinants.

## Funding and conflict of interest

This study was partially funded by the Department of Obstetrics and Gynecology, section of Reproductive Medicine, University Medical Center Groningen. None of the authors have conflicts of interest to declare.

Overadjustment is defined inconsistently. This term is meant to describe control (eg, by regression adjustment, stratification, or restriction) for a variable that either increases net bias or decreases precision without affecting bias. We define overadjustment bias as control for an intermediate variable (or a descending proxy for an intermediate variable) on a causal path from exposure to outcome. We define unnecessary adjustment as control for a variable that does not affect bias of the causal relation between exposure and outcome but may affect its precision. We use causal diagrams and an empirical example (the effect of maternal smoking on neonatal mortality) to illustrate and clarify the definition of overadjustment bias, and to distinguish overadjustment bias from unnecessary adjustment. Using simulations, we quantify the amount of bias associated with overadjustment. Moreover, we show that this bias is based on a different causal structure from confounding or selection biases. Overadjustment bias is not a finite sample bias, while inefficiencies due to control for unnecessary variables are a function of sample size.

## CRediT authorship contribution statement

**Talita Honorato-Rzeszewicz:** Writing – review & editing, Writing – original draft, Formal analysis, Conceptualization. **Annemieke Hoek:** Writing – review & editing, Supervision, Conceptualization. **Maaike L. Haadsma:** Writing – review & editing, Supervision, Conceptualization. **Henk Groen:** Writing – review & editing, Writing – original draft, Methodology, Formal analysis, Conceptualization.

## Declaration of competing interest

The authors declare that they have no known competing financial interests or personal relationships that could have appeared to influence the work reported in this paper.

## Data Availability

The authors do not have permission to share data.
